# Transperitoneal robot-assisted partial nephrectomy: a comparison of operative and oncological outcomes between posterior and anterolateral tumours

**DOI:** 10.1007/s11255-025-04372-x

**Published:** 2025-01-14

**Authors:** Frederik F. Thomsen, Rasmus D. Petersson, Katrine S. Schou-Jensen, Badal S. Rashu, Malene H. Niebuhr, Nessn H. Azawi

**Affiliations:** 1 Department of Urology, Copenhagen University Hospital, Herlev and Gentofte Hospital, Herlev, Denmark; 2 Department of Urology, Zealand University Hospital, Roskilde, Denmark; 3 Institute of Clinical Medicine, Copenhagen University, Copenhagen, Denmark

**Keywords:** Renal cell carcinoma, Partial nephrectomy, Tumour location, Posterior, Oncological, Complication

## Abstract

**Objective:**

To compare operative and oncological outcomes, as well as the risk of postoperative complications in patients who underwent transperitoneal robot-assisted partial nephrectomy (RAPN) for renal tumours located either posteriorly or anterolaterally.

**Methods:**

Retrospective, consecutive study including 451 patients who underwent transperitoneal RAPN for non-metastatic, localised renal tumours from May 2016 to April 2023. Operative data included duration of the procedure, warm ischaemia time, and blood loss; oncological data included surgical margins and recurrence; and 90-day postoperative complications were classified according to the Clavien-Dindo classification.

**Results:**

In total, 140 (31%) patients had tumours with a posterior location. The median follow-up was 3.3 (IQR 1.8–5.0) years. There were no differences in operative outcomes or length of hospital stay between the two groups. Positive surgical margins were recorded in 9% of the patients with posterior tumours compared to 7% of patients with anterolateral tumours, *p* = 0.60. The estimated probability of recurrence-free survival at 5 years was 95.2% (95% CI 87.4–98.2) for patients with posterior tumours and 96.7% (95% CI 92.3–98.6) for patients with anterolateral tumours, *p* = 0.4. Patients with posterior tumours had a similar risk of any complication (OR 1.24 [95% CI 0.80–1.91]) and CD ≥ III (OR 0.73 [95% CI 0.28–1.67]) compared to patients with anterolateral tumours.

**Conclusion:**

This study found that patients with posterior tumours had longer operating times and hospital stays following transperitoneal RAPN compared to those with anterolateral tumours but without increased complications or poorer oncological outcomes.

**Graphical abstract:**

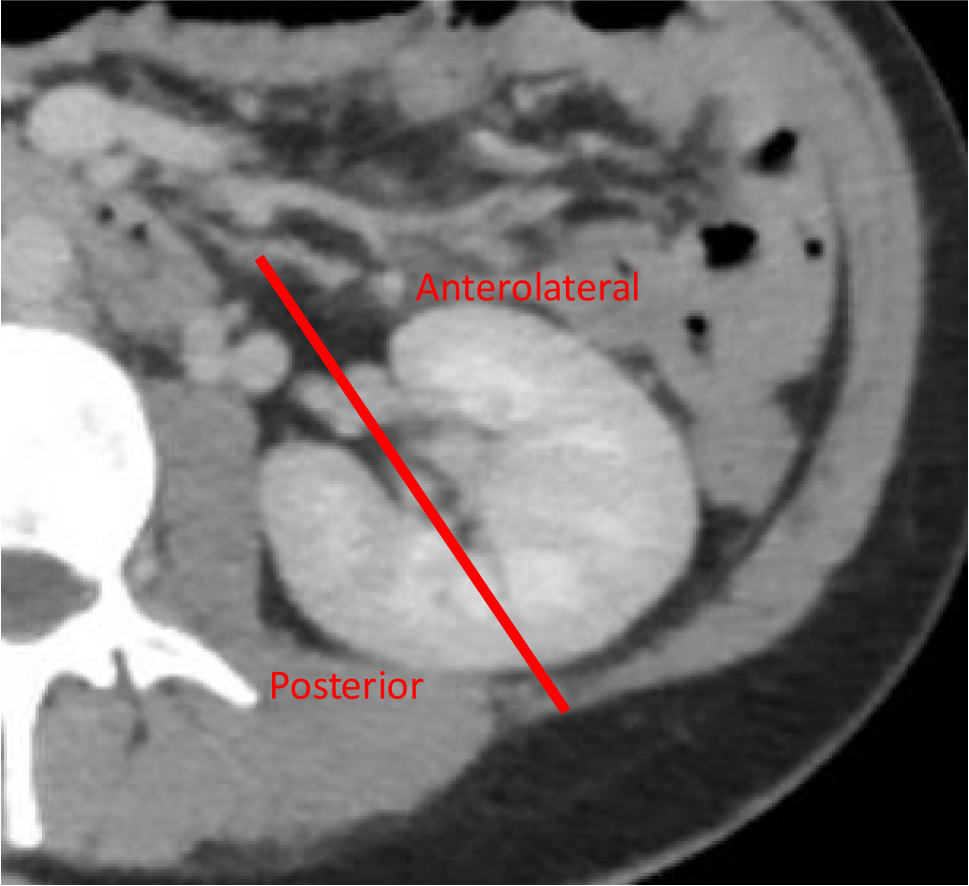

## Introduction

Renal cell carcinoma (RCC) represents approximately 3% of all cancers [1], and the incidence has increased over the last 20 years [2] – mainly due to incidental findings of small renal masses [3–5]. The preferred curative treatment modality for T1 renal tumours is partial nephrectomy (PN) because it better preserves renal function compared to radical nephrectomy without compromising the oncological outcomes [6, 7].

Robot-assisted laparoscopic PN (RAPN) can be performed with either transperitoneal or retroperitoneal access [8]. The choice of access is open for debate and is often surgeon-dependent and the transperitoneal access for PN has traditionally been used in Denmark [9–12]. However, transperitoneal RAPN for posterior tumours necessitates more extensive kidney mobilisation to achieve optimal access to the tumour and often pose challenges in terms of renorrhaphy, particularly in cases of higher complexity. [13].

Since the introduction of retroperitoneal access for RAPN in 2023 in our department, our focus has shifted towards understanding its implications more clearly. Specifically, we wanted to investigate whether patients with posteriorly located tumours, i.e. tumours more easily reachable via retroperitoneal access [13], experienced less favourable perioperative or oncological outcomes or a higher rate of complications when undergoing transperitoneal RAPN, compared to patients with anterolateral tumours.

Thus, the objective of this study was to compare operative and oncological outcomes, as well as the risk of postoperative complications, in patients who underwent transperitoneal RAPN for posterior tumours compared to patients with anterolateral tumours.

## Material and methods

Retrospectively and consecutive study including all patients who underwent transperitoneal RAPN for non-metastatic, localised renal tumours from May 2016 to April 2023. Subsequent electronic patient chart review was performed. The following preoperative data were extracted: age, gender, Charlson Comorbidity Index [CCI] [14], American Society of Anaesthesiologists classification [ASA] [15], Eastern Cooperative Oncology Group [ECOG] performance status [16], body mass index [BMI], smoking status, kidney function [eGFR], tumour size, tumour location [anterior, lateral, posterior], and Preoperative Aspect and Dimensions Used for an Anatomical (PADUA) classification score [17]. The following operative data were extracted: duration of the procedure, warm ischaemia time, blood loss, surgeon. Six primary surgeons performed 419/451 (93%) of the procedures. The remaining procedures were performed by visiting surgeons or by trainee consultant surgeons supervised by one of the primary surgeons (grouped as surgeon 7). Following follow-up data were extracted: complications within 90 days classified according to the Clavien-Dindo classification (CD) [18], and recurrence date in the event.

The da Vinci Si system (Intuitive Surgical, Inc.) was used in 437/451 (97%) of cases. The 14 remaining cases were performed with the da Vinci X system (Intuitive Surgical, Inc.).

The study received ethical and legal approval from the regional centre for register research of the Capital Region of Denmark according to Danish law (journal number: R-23018007).

Descriptive statistics were applied. Median follow-up time was calculated using the reverse Kaplan–Meier method [19]. Retrospectively, patients were classified into two groups based on the tumour location defined preoperative CT scan (Figure): “anterolateral” tumours and “posterior” tumours. Categorical variables were analysed using the Chi-square test, while continuous variables were assessed using the Wilcoxon test. Recurrence-free survival was estimated using Kaplan–Meier curves, and Log-Rank test was applied to compare recurrence rates between the groups. Trifecta outcome was defined as: no perioperative complications, negative surgical margins, and less than 15% decline in estimated glomerular filtration rate (eGFR) one year after RAPN. Odds ratios (OR) with 95% confidence intervals (CI) for postoperative complications were determined through uni- and multivariate logistic regression analyses controlling for pre- and perioperative variables. The model investigating any complication included the following preoperative variables: age (< 55, 55–64, 65–74, > 75), CCI (0, 1, ≥ 2), ASA (1, 2, ≥ 3), PADUA score (6–7, 8–9, 10–13), and perioperative variables: duration of the procedure (quartiles), warm ischaemia time (quartiles), blood loss (quartiles), and surgeon. For the model investigating a CD ≥ 3 complication included: CCI (0, 1, ≥ 2) and blood loss (quartiles). All tests were two-sided, and the significance level was set to *p* < 0.05. Statistical analysis was performed with R version 4.0.0 (R Foundation for Statistical Computing, Vienna, Austria).

## Results

Baseline characteristics of the 451 patients included are presented in Table 1. In total, 140 (31%) patients had posteriorly located tumours, and there were no significant differences in baseline characteristics between these patients and patients with anterolaterally located tumours. The median follow-up was 3.3 (IQR 1.8–5.0) years with no patients lost to follow-up.

**Table 1:** Baseline characteristics of 451 patients who underwent partial nephrectomy for localised renal masses in May 2016 – April 2023

	Posterior tumours		Anterior or lateral tumours		*p*
	*n* = 140		*n* = 311		
	*n*	%	*n*	%	
*Gender*					0.86*
Female	48	34	104	33	
Male	92	66	207	67	
*Age, years*					
Median (IQR)	64.5 (56–73)		63 (54–70)		0.27**
*Body Mass Index*					
Median (IQR)	27 (24–30)		27 (24–30)		0.58**
Missing	6		14		
*Charlson Comorbidity score*					0.40*
0	85	61	187	60	
1	32	23	85	27	
≥ 2	23	16	39	13	
*Performance status*					0.15*
0	86	61	220	71	
1	45	32	74	24	
2	8	6	16	5	
Missing	1	1	1	1	
*ASA score*					0.07*
1	23	16	46	15	
2	70	50	190	61	
≥ 3	47	34	75	24	
*Tobacco use*					0.65*
Never	59	42	116	37	
Former	32	23	77	25	
Active	46	33	109	35	
Missing	3	2	9	3	
*Previous abdominal surgery*					0.67*
No	100	71	216	69	
Yes	40	29	95	31	
Open	23	16	60	19	
Laparoscopic	17	12	35	11	
*Tumour size, mm*					0.16**
Median (IQR)	26.5 (20–37)		30 (20–40)		
*PADUA score*					0.72**
Median (IQR)	7.5 (7–9)		7 (7–9)		
6–7	70	50	172	55	
8–9	56	40	102	33	
10–13	14	10	37	12	
*Surgeon*					0.15*
1	34	24	90	29	
2	37	26	83	27	
3	23	16	40	13	
4	6	4	33	11	
5	17	12	22	7	
6	12	9	22	7	
7	11	8	21	7	

*Chi-sq

**Wilcox test

Patients with posterior tumours had a longer median duration of the procedure of 185 (IQR 161–223) min compared to 167 (IQR 142–196) min in patients with anterolateral tumours, *p* < 0.01. Comparable numbers for median warm ischaemia time were 15 (IQR 12–19) min and 15 (IQR 12–18) min, *p* = 0.55, median perioperative blood loss was 100 (IQR 50–200) ml in both groups, *p* = 0.06, and median length of stay at the hospital was 3 (IQR 2–3) days in the anterolateral group and 3 (IQR 3–5) days in the posterior group, *p* = 0.03.

An overview of peri- and postoperative complications can be seen in Table 2. Patients with posterior tumours did not have an increased risk of any complication (OR 1.24 [95% CI 0.80–1.91]) or of a CD ≥ III (OR 0.73 [95% CI 0.28–1.67]) compared to patients with an anterolateral tumour, Table 3. Adjusting for pre- or perioperative variables did not change the outcome.

**Table 2:** Peri- and postoperative complications, surgical margin, loss in eGFR and Trifecta outcomes following transperitoneal robot-assisted laparoscopic partial nephrectomy for localised renal masses stratified on tumour location

	Posterior tumours		Anterior or lateral tumours	
	*n* = 140		*n* = 311	
	*n*	%	*n*	%
*Perioperative complications*				
Spleen lesion			4	1
Diaphragm lesion	1	0.7	1	0.3
Small intestine injury	1	0.7		
*Postoperative complications*				
No	95	68	225	72
Yes	45	32	86	28
*Clavien-Dindo*				
I	12	9	12	4
II	26	19	53	17
IIIa	1	0.7	3	1
IIIb	5	4	16	5
IVb	-	-	1	0.3
V	1	0.7	1	0.3
*Surgical margin*				
Negative	119	85	275	88
Positive	13	9	22	7
Indeterminant	8	6	14	5
*Loss in eGFR after 1 year*	*n* = 131		*n* = 294	
Less than 15% decline	87	66	231	79
More than 15% decline	44	34	63	21
Trifecta*	*n* = 131		*n* = 294	
Yes	73	56	202	69
No	58	44	92	31

*eGFR* estimated glomerular filtration rate

*No perioperative complications, negative surgical margins, and less than 15% decline in eGFR

**Table 3:** Uni- and multivariable logistic regression analyses of odds for experiencing a postoperative complication adjusting for pre- and perioperative variables

	Univariate Logistic regression				Multivariable logistic regression adjusting for preoperative variables				Multivariable logistic regression adjusting for perioperative variables			
	Any complication		CD ≥ 3		Any complication		CD ≥ 3		Any complication		CD ≥ 3	
	OR	95% CI	OR	95% CI	OR	95% CI	OR	95% CI	OR	95% CI	OR	95% CI
*Tumour location*												
Anterior or lateral	Ref				Ref		Ref		Ref		Ref	
Posterior	1.24	0.80–1.91	0.73	0.28–1.67	1.13	0.72–1.76	0.67	0.26–1.56	1.14	0.65–1.98	0.64	0.24–1.50
*Age*												
< 55	Ref		Ref		Ref							
55–64	1.70	0.95–3.09	4.23	1.06–28.2	1.61	0.89–2.98						
65–74	1.57	0.90–2.81	4.79	1.27–31.2	1.38	0.75–2.55						
≥ 75	2.15	1.07–4.31	5.05	1.05–36.0	1.87	0.90–3.89						
*Gender*												
Female	Ref		Ref									
Male	0.96	0.63–1.48	1.93	0.81–5.34								
*Charlson comorbidity score*												
0	Ref		Ref		Ref		Ref					
1	0.72	0.43–1.19	0.82	0.26–2.21	0.58	0.31–1.04	0.81	0.26–2.18				
≥ 2	2.09	1.18–3.67	3.13	1.25–7.52	1.43	0.78–3.01	3.21	1.27–7.74				
*Performance status*												
0	Ref		Ref									
1	1.46	0.92–2.30	2.12	0.92–4.81								
2	1.67	0.68–3.90	2.98	0.65–10.0								
*ASA*												
1	Ref		Ref		Ref							
2	1.43	0.78–2.77	0.97	0.29–4.39	1.33	0.70–2.65						
≥ 3	1.89	0.97–3.83	2.85	0.89–12.7	1.68	0.74–3.91						
*Body mass index*												
< 25	Ref		Ref									
25–29	0.68	0.41–1.13	0.60	0.22–1.52								
≥ 30	0.83	0.50–1.37	0.53	0.18–1.39								
*Smoking*												
Never	Ref		Ref									
Active smoker	0.97	0.56–1.67	1.90	0.66–5.57								
Former smoker	1.54	0.96–2.48	2.19	0.88–5.99								
*Tumour size*												
q1	Ref		Ref									
q2	1.49	0.88–2.56	1.55	0.50–5.25								
q3	0.86	0.41–1.72	1.66	0.40–6.50								
q4	1.82	1.05–3.19	2.87	1.01–9.34								
*PADUA*												
6–7	Ref		Ref		Ref							
8–9	1.34	0.86–2.07	1.02	0.43–2.31	1.28	0.82–2.02						
10–13	1.03	0.51–1.99	0.95	0.21–3.01	1.04	0.50–2.06						
*Duration procedure*												
q1	Ref		Ref						Ref			
q2	1.67	0.84–3.36	2.65	0.55–18.8					1.38	0.66–2.92		
q3	1.39	0.69–2.83	2.07	0.39–15.2					0.93	0.42–2.03		
q4	3.00	1.57–5.93	6.27	1.62–41.3					1.56	0.71–3.50		
*Warm ischemia time*												
q1	Ref		Ref						Ref			
q2	1.78	0.98–3.28	2.44	0.75–9.38					1.63	0.77–3.45		
q3	1.70	0.94–3.10	2.59	0.82–9.79					1.57	0.73–3.40		
q4	2.55	1.41–4.70	2.04	0.57–8.18					2.57	1.18–5.71		
*Blood loss*												
q1	Ref		Ref						Ref		Ref	
q2	0.81	0.41–1.52	3.47	0.83–17.3					0.61	0.27–1.33	3.57	0.85–17.8
q3	2.42	1.35–4.35	5.94	1.60–28.2					1.79	0.88–3.63	6.29	1.69–30.0
q4	2.94	1.74–5.01	8.15	2.55–36.2					2.08	1.05–4.14	8.34	2.60–37.1
*Surgeon*												
1	Ref		Ref						Ref			
2	0.68	0.38–1.21	0.58	0.15–1.96					0.69	0.34–1.40		
3	1.02	0.52–1.95	1.44	0.41–4.71					0.94	0.43–2.04		
4	1.64	0.77–3.44	1.39	0.29–5.30					1.72	0.67–4.40		
5	0.71	0.29–1.58	1.39	0.29–5.30					0.55	0.18–1.52		
6	1.65	0.74–3.59	2.23	0.55–7.89					2.03	0.71–5.72		
7	0.92	0.37–2.12	1.11	0.16–4.89					0.65	0.22–1.77		

There were no major differences in pathological outcomes between the two groups. In total, 128 (91%) of patients with posterior tumours had RCC, of which 91% were pT1a and 64% were ccRCC. Comparable numbers in patients with anterolateral tumours were 288 (93%), of which 85% were pT1a and 60% were ccRCC. 13 (9%) patients with posterior tumours had positive surgical margins compared to 22 (7%) of patients with anterolateral tumours, *p* = 0.60, Table 2. One year after RAPN 87/131 (66%) of patients with posterior tumours had a less than 15% decline in eGFR compared to 231/294 (79%) of patients with anterolateral tumours, *p* = 0.01, Table 2. All but one patient had an eGFR higher than 30 mL/min/m2 one year after RAPN. The patient in question had a preoperative eGFR of 16 mL/min/m2. In total, 56% of patients with posterior tumours fulfilled the Trifecta criteria compared to 69% of patients with anterolateral tumours, *p* = 0.01, Table 2.

The estimated probability of recurrence-free survival at 5 years was 95.2% (95% CI 87.4–98.2) for patients with posterior tumours and 96.7% (95% CI 92.3–98.6) for patients with anterolateral tumours, *p* = 0.4.

## Discussion

In this retrospective, consecutive study of 451 patients who underwent RAPN, we found that the tumour location did not significantly impact the perioperative or oncological outcomes, nor the risk of postoperative complication or risk of recurrence. We did, however, find that the procedure time in patients with posterior tumours had a median extension of 18 min (i.e. 11% longer) compared to patients with anterolateral tumours, 56% patients with posterior tumours fulfilled the Trifecta criteria compared to 69% of patients with anterolateral tumours, and that some patients with posterior tumours (median 3 [IQR 3–5] days) had a longer admission compared to patients with anterolateral tumours (median 3 [IQR 2–3] days).

Two previous studies have compared posterior tumours with anterolateral tumours in RAPN performed with transperitoneal access. Harris et al. compared surgical outcomes between 92 patients with posterior tumours and 168 patients with anterolateral tumours [20]. They found no difference in warm ischaemia time, duration of the procedure, blood loss, positive margins or postoperative complications between these groups. Similarly, Takagi et al. found no difference in operative outcomes in a propensity score-matched comparison of 82 patients with posterior and 82 patients with anterolateral tumours who underwent transperitoneal RAPN [21]. The current study found that fewer patients with posterior tumours fulfilled the Trifecta criteria one year postoperatively, primarily because more patients with posterior tumours experienced a loss of eGFR greater than 15% one year after RAPN. The long-term implications of the greater loss of eGFR in patients with posterior tumours are unknown, but could potentially result in a higher incidence of chronic kidney disease in this group.

Two systematic reviews comparing RAPN with transperitoneal or retroperitoneal access found that the retroperitoneal access had similar operative and oncological outcomes to the transperitoneal access [22, 23]. Thus, based on the current evidence, the choice of access for RAPN is surgeon-dependent.

The benefits of transperitoneal access include more recognisable landmarks, more space to mobilise the kidney, and most surgeons have more experience with this access because it is used for other urological procedures such as radical nephrectomy, nephroureterectomy, and pyeloplasty [24–29]. The learning curve for transperitoneal RAPN is generally short, with approximately 20 cases needed to achieve a console time of less than 100 min, 77 cases are needed to reach a plateau of the procedure time, and 26 cases are needed for an averaged warm ischaemia time of less than 15 min [30].

The retroperitoneal access might be advantageous in patients with posterior upper pole or perihilar tumours, where previous studies have found shorter operating time and length of stay in these patients compared to the transperitoneal access [22, 23]. Although retroperitoneal access offers easier access to posterior tumours, this access is in these authors’ opinion, more challenging and requires a greater experience and expertise compared to transperitoneal access. However, no study has to date investigated the learning curve for RAPN with retroperitoneal access. The retroperitoneal access likely also offers a benefit in patients with previous extensive abdominal surgery, where this access would circumvent bowel adhesions. Thus, mastering both accesses seems beneficial – considering their relative benefits in different cases.

One consideration that may influence the choice of surgical access is the length of hospital stay, which in the current study was longer for patients with posteriorly located tumours. However, previous studies have reported comparable length of hospital stays following RAPN, regardless of whether the procedure was performed transperitoneally or retroperitoneally [31–33], suggesting a potential cost–benefit to utilizing the retroperitoneal access for posterior tumour. Additionally, advancements in robotic systems, which can assist in RAPN [34] could further enhance the cost-effectiveness of these procedures [35]. Thus, future studies, preferably prospective, are needed to investigate the impact of anatomical location and robotic systems with regard to RAPN for different tumour locations.

The main limitation of the study is its retrospective design, which could introduce biases and confounders that we were unable to control for, as well as the underreporting of postoperative complications. To mitigate these limitations, we performed multivariable regression analyses, adjusting for pre- and perioperative variables. The strengths of the study are complete follow-up and the fact that postoperative care was similar for the entire cohort as they were all treated, at the same centre.

## Conclusion

This study of RAPN performed with transperitoneal access found that the operating time and length of hospital stay for patients with posterior renal tumours were increased compared to patients with anterolateral tumours and fewer fulfilled the Trifecta criteria. However, this did not translate into an increased risk of complications or poorer oncological outcomes.

## Data Availability

No datasets were generated or analysed during the current study.
